# Exploring mentorship as a strategy to build capacity for knowledge translation research and practice: protocol for a qualitative study

**DOI:** 10.1186/1748-5908-4-55

**Published:** 2009-08-19

**Authors:** Anna R Gagliardi, Laure Perrier, Fiona Webster, Karen Leslie, Mary Bell, Wendy Levinson, Ori Rotstein, Ann Tourangeau, Laurie Morrison, Ivan L Silver, Sharon E Straus

**Affiliations:** 1 Toronto General Research Institute, University Health Network, Toronto, Canada; 2 Continuing Education and Professional Development, Faculty of Medicine, University of Toronto, Toronto, Canada; 3 Centre for Health Services Sciences, Sunnybrook Health Sciences Centre, Toronto, Canada; 4 Centre for Faculty Development, St. Michael's Hospital, Toronto, Canada; 5 Department of Medicine, Faculty of Medicine, University of Toronto, Toronto, Canada; 6 Department of Surgery, St. Michael's Hospital, Toronto, Canada; 7 Faculty of Nursing, University of Toronto, Toronto, Canada; 8 Emergency Medicine and Critical Care, St. Michael's Hospital, Toronto, Canada; 9 General Internal Medicine, St. Michael's Hospital, Toronto, Canada

## Abstract

**Background:**

Research funders, educators, investigators and decision makers worldwide have identified the need to improve the quality of health care by building capacity for knowledge translation (KT) research and practice. Peer-based mentorship represents a vehicle to foster KT capacity. The purpose of this exploratory study is to identify mentoring models that could be used to build KT capacity, consult with putative mentee stakeholders to understand their KT mentorship needs and preferences, and generate recommendations for the content and format of KT mentorship strategies or programs, and how they could be tested through future research.

**Methods:**

A conceptual framework was derived based on mentoring goals, processes and outcomes identified in the management and social sciences literature, and our research on barriers and facilitators of academic mentorship. These concepts will inform data collection and analysis. To identify useful models by which to design, implement and evaluate KT mentorship, we will review the social sciences, management, and nursing literature from 1990 to current, browse tables of contents of relevant journals, and scan the references of all eligible studies. Eligibility screening and data extraction will be performed independently by two investigators. Semi-structured interviews will be used to collect information about KT needs, views on mentorship as a knowledge sharing strategy, preferred KT mentoring program elements, and perceived barriers from clinician health services researchers representing different disciplines. Qualitative analysis of transcripts will be performed independently by two investigators, who will meet to compare findings and resolve differences through discussion. Data will be shared and discussed with the research team, and their feedback incorporated into final reports.

**Discussion:**

These findings could be used by universities, research institutes, funding agencies, and professional organizations in Canada and elsewhere to develop, implement, and evaluate mentorship for KT research and practice. This research will establish a theoretical basis upon which we and others can compare the cost-effectiveness of interventions that enhance KT mentorship. If successful, this program of research may increase knowledge about, confidence in, and greater utilization of KT processes, and the quality and quantity of KT research, perhaps ultimately leading to better implementation and adoption of recommended health care services.

## Introduction

### Knowledge translation

Health care is often not delivered according to recommendations that are based on the best available research. Numerous population-based studies in Canada, Australia, the United Kingdom and United States demonstrate low compliance with guidelines for preventive, acute, and chronic care services [1-6]. Knowledge translation (KT) refers to an iterative process for improving health care delivery and associated outcomes by promoting research utilization in decision making. The 'knowledge-to-action' cycle involves synthesizing knowledge, interacting with target users to assess needs and identify barriers, using that information to tailor knowledge products and select implementation strategies, and ongoing monitoring to evaluate impact [7]. Health professionals have professed that they are unfamiliar with the concept and practice of KT, which may explain why research findings and knowledge products such as practice guidelines continue to be passively disseminated [5-11].

Comprehensively implementing the findings of clinical and health services research into practice is one dimension of KT that can improve health care appropriateness and outcomes (KT practice). Another important dimension is the conduct of research that identifies barriers of appropriate practice, and evaluates interventions to improve the organization and delivery of care (KT research). The need to foster KT research in nursing and primary care was recognized in Australia and the United Kingdom by reviewing the literature and consulting with health professionals [12-15]. KT was prioritized among practitioners and teachers of emergency medicine from 16 countries [16]. They underscored the need to form linkages with KT scientists to foster and support the conduct of KT research and practice [17-19]. Interviews with individuals from 33 research funding agencies worldwide also revealed the need to better implement research knowledge into practice by increasing our understanding and practice of KT [20,21]. Others have investigated mechanisms by which to increase general or cross-disciplinary research capacity, but these efforts have largely focused on increasing infrastructure and resources [22-24].

### Mentorship

Educational and social learning theories provide a basis upon which to develop mechanisms that foster capacity for KT research and practice. Principles developed by Knowles, Candy, Bandura, and Schon suggest that professionals should be active contributors in the educational process so that learning is work-situated, or shaped by their knowledge and experience, and teachers or role models should facilitate learning by providing guidance, support, and constructive feedback [25]. Social interaction is also a powerful facilitator of learning and behaviour change. Personal contact with researchers has been repeatedly cited by health professionals as the factor most influencing their decisions about adopting new practices or programs [26-28].

Coaching or mentoring is an interactive, facilitative process meant to promote learning and development that is based on educational and social learning theories [29]. Mentoring has been studied largely within the context of large corporations where it is used for training and succession planning [30]. While there is no universal consensus on any particular definition or form, mentoring is typically thought of as a hierarchical relationship between a senior and junior organizational member to help the protégé advance with the organization [29]. Seminal research by Kram found that mentoring consists of support for both career (sponsorship, exposure and visibility, coaching, protection, challenging) and psychosocial (role modeling, acceptance and confirmation, counseling, friendship) development, and typically proceeds through four stages: initiation, cultivation, separation, and redefinition [31]. Significant benefits are associated with mentorship. Protégés receive more promotions, have higher salaries, experience less stress and conflict, are more satisfied with their jobs and careers, and are less likely to leave their organizations compared with non-protégés [32,33]. These positive outcomes are associated with both formal (matches made by a third party) and informal (self-initiated) mentorship, and are sustained longitudinally compared with those not mentored [33-35]. Mentors also derive benefit from mentoring, including satisfaction from helping others, creation of free time for alternate pursuits, organizational recognition or reward, and improved job performance through exposure to new ideas [36].

Early work by Kram and others noted some disadvantages of traditional hierarchical mentorship, and recognized that other forms of mentoring may be more suitable for different types of learning and development [37,38]. In particular, peer mentoring could be used to help individuals develop new skills, encourage continuous learning and cross-disciplinary experiences, make contact with internal and external experts, and stay informed of changes and developments in one's own, or other professions [37]. Kram interviewed 15 early-, middle- and late-career managers from one organization and two peer mentors identified by each [38]. Mentors assumed different roles of information peer, collegial peer, or special peer on a continuum with increasing levels of psychosocial support to accompany more basic job-specific counseling. While there appears to be substantial research conducted in the 1980s and 1990s describing corporate, hierarchical mentorship functions and outcomes, limited empirical research has examined how mentorship can be most effectively designed and implemented for non-corporate applications, and based on alternative forms such as peer mentorship.

### Mentorship in health care

In health care, mentoring has been used for teaching student and novice nurses about clinical practice [39-43]. A large proportion of nurses report having one or more mentors who serve a variety of formal and informal roles [44]. Analysis of 82 articles published from 1977 to 1994 found that nursing mentorship was associated with greater job satisfaction and academic achievement [45]. Interviews with, and surveys of mentor and protégé nurses emphasized the need for greater clarity of objectives, and the development of strategies to identify and train mentors [46-48]. Mentoring is also recognized within the nursing literature as an important mechanism for research training in both university and clinical settings but this has not been thoroughly described or evaluated [49,50].

Academic mentoring has been used to promote professional advancement and research success among health professional faculty. SES conducted a systematic review to assess the evidence about the use and outcomes of academic mentorship in health care. Among 39 studies published between 1985 and 2006, there were no randomized trials of mentoring interventions [51]. Less than 20% of junior faculty had a mentor, and women reported greater difficulty finding mentors compared with male colleagues. SES subsequently interviewed 28 clinician scientists and mentors at two Alberta universities to describe the characteristics of successful academic mentoring relationships [52]. While considered important by all participants, preferences differed on the formality, structure, and evaluation of the mentoring process. Mentees described difficulty identifying and selecting mentors, and establishing the parameters of the mentoring relationship. Participants differentiated mentoring goals and functions. For example, career mentoring was thought to include guidance for achieving career milestones and promotion, navigating local politics, and maintaining work-life balance. Participants thought that research mentoring included identification of sources of funding, review of grants and manuscripts, and facilitating linkages with collaborators. This study involved a small number of physician investigators at two academic institutions, so further studies should explore and compare these views with those of physician and non-physician investigators at other sites.

Mentorship between faculty and medical students can facilitate the transfer of technical skill and tacit knowledge about ethics, values, professionalism, and the art of medicine, but research suggests there are few such programs, so we lack information on how medical education mentoring is structured and implemented, barriers to its utilization, and how it could be improved [53]. A review of the literature on mentoring for medical students identified nine eligible articles published between 1961 and 2003 [54]. Program goals varied widely, including orientation to student life and health care facilities, recruitment to general practice, and introduction to research methods. Structure and duration also varied. Details regarding mentor-mentee matching and outcomes were absent. A systematic review of studies evaluating the effect of mentoring on career choice found that less than 50% of medical students reported having a mentor [51]. Those that did said it had an important influence on personal development and career choice.

### Mentorship for KT practice and research

Research funders, educators, investigators, and decision makers worldwide have identified the need to improve the quality of health care by building capacity for KT research and practice. To build this capacity we need strategies or programs that would involve KT researchers in helping investigators with an interest in, but little or no knowledge of KT to undertake research that evaluates interventions designed to overcome barriers of appropriate care delivery (KT research), and apply KT methods so that their clinical or health services research findings, or knowledge synthesis products are utilized by target stakeholders (KT practice). Peer-based mentorship represents a promising mechanism for sharing knowledge and warrants further investigation as a vehicle to foster KT research and practice. We are not aware of published studies that developed, implemented, or evaluated mentorship as an intervention to build capacity for KT research and practice among physician and non-physician clinical and health services researchers as putative mentees. The views of KT researchers who might be mentors will be explored in a separate study so that we can elicit their perspectives based on the findings of this study. Collective findings will be used in a series of future studies to develop, pilot-test, and then more rigorously evaluate KT mentorship programs, including component tools and strategies. The purposes of this exploratory research study are to: identify mentoring models that could be used to build capacity for KT research and practice; consult with putative mentee stakeholders to understand their KT mentorship needs and preferences; and, based on these findings, generate recommendations for the content and format of KT mentorship strategies or programs, and how they could be tested through future research.

### Theoretical framework

**Figure 1:**
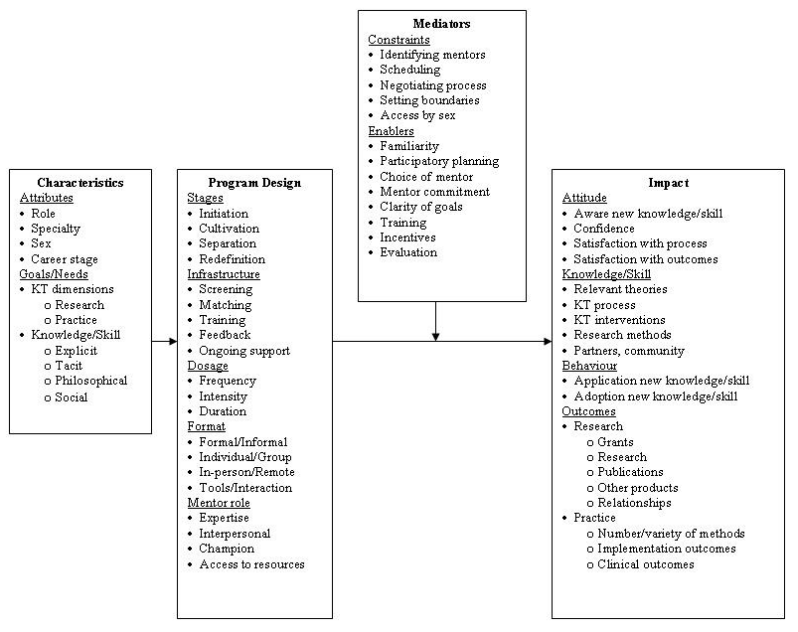
**Conceptual framework of factors that influence preferences, design and outcomes of mentorship**.

There is no single theory or model that describes the processes, outcomes, and factors influencing mentorship therefore a conceptual framework was derived to inform data collection and analysis (Figure 1). This was based on a review by Karcher of the educational and psychology literature that identified elements of program design that might influence mentoring outcomes (goals, delivery, structure, content, mediators) [29]. Additional goals and processes of mentoring were described by Kram (hierarchical or peer, career or psychosocial, stages of initiation, cultivation, separation, and redefinition) [34]. SES identified barriers and facilitators of mentoring in previous research (identifying/securing mentors, sex, familiarity, choice of mentor, mentor commitment, scheduling, clarity of goals, negotiating process, preferences, training, stage-specific evaluation, incentives) [52]. We also reviewed the literature on opinion leaders and knowledge brokers, and theories relevant to behaviour change that involved facilitation to describe the role of a mentor [55-58].

## Methods

### Mentorship models

To identify useful models by which to design, implement, and evaluate KT mentorship, we will conduct a scoping review of the literature as described by Arksey [59]. Because the previous review of the medical literature on academic mentorship found little evidence to guide the development of research mentoring programs [51], and that review is currently being updated by one of the co-investigators (SES), this review will focus on empirical studies of professional mentoring in the social sciences, management, and nursing literature. Searches of indexed sources will be executed for the years 1990 to current to encompass a nearly 20-year span of research. To augment these searches, we will examine five years of tables of contents for select journals that appear to publish research on mentorship, and scan the references of all eligible studies. Preliminary selection criteria (to be further informed by nature of identified studies) include quantitative (meta-analyses, questionnaire surveys, observational studies, randomized trials) and qualitative (reviews/conceptual analyses, interviews, focus groups) studies published in English from 1990 to current that focus on developing or evaluating mentorship programs.

Eligibility screening and data extraction will be performed independently by two investigators using a data extraction form that reflects elements of the conceptual framework (Figure 1). Most details will be noted on the form by checking the appropriate box. Relevant qualitative details will be highlighted in the article, copied, and attached to the data extraction form. Methods for extracting and describing eligible literature, which is likely to include both qualitative and quantitative studies, will be guided by Mays, who suggests that 'narrative synthesis' of information from various types of studies is appropriate for developing knowledge at an early stage in policy development [60]. This involves direct reporting of findings rather than quantitative or thematic synthesis. Study quality will be assessed using criteria relevant to study design, but will not be used to exclude studies [61,62].

Data will be tabulated and examined to describe the quantity, design, and quality of studies. The nature of mentorship programs will be discussed according to the modified conceptual framework, including goals, delivery, structure, content, mediators, and outcomes. Raw and synthesized data will be shared and discussed with the research team, and their feedback incorporated into final reports. The final product will be two-fold: 1) a report describing different mentoring models, the degree to which they have been evaluated, their apparent effectiveness, and possible applicability to KT mentoring tasks such as identifying sources of funding, review of grants and manuscripts, creating linkages with collaborators, transfer of research skills, confidence-building, and encouraging a focus on practice-relevant research; and 2) recommendations for research where gaps in knowledge are revealed.

### KT mentorship needs and preferences

Semi-structured interviews will be used to collect information about KT needs for research and practice (goals), views on mentorship as a knowledge-sharing strategy, preferred KT mentoring program elements (delivery, structure, content), and perceived mediators of KT mentorship (constraints, enablers) [63]. Standard methods of grounded qualitative research will be used for sampling and analysis [64]. Ten consenting candidates (convenience sampling) will be recruited from each of the faculty of nursing and three departments in the faculty of medicine representing physician and non-physician health services researchers who differ by career stage (junior, senior), and sex (male, female), for a minimum total of 40 interviews (purposive sampling). Detailed information from representative, rather than a large number of cases, is needed in qualitative research. Sampling is concurrent with data collection and analysis (grounded approach), and proceeds until no further unique themes emerge from successive interviews (saturation). If thematic saturation is not achieved within sampling subcategories (career stage, sex), further interviews will be pursued.

Unique themes will be identified in an inductive manner through iterative stages according to standard methods of qualitative analysis [65,66]. Transcribed narrative will be read to identify, define, and organize themes relevant to study objectives (open coding). A log will be maintained of emerging thematic codes, their definition, sample data illustrating application of that code, and an account of decisions related to that code. The growing narrative will repeatedly be reviewed (constant comparative technique) to identify all instances of the coding framework, as well as all instances that do not match the framework, and determine whether and how to expand or merge thematic codes (axial coding). Qualitative analysis will be conducted manually, without the assistance of software, which cannot perform analytic tasks. To improve the reliability of these findings the coding framework, code book and narrative will be reviewed by a second investigator. The two will meet to compare findings and achieve consensus through discussion. Text representing KT mentorship needs and preferences will be tabulated by theme, faculty and department, professional role, sex, career stage, and perceived mediators. The final product will be a report with summary tables highlighting the elements of preferred KT mentoring programs according to stakeholder needs and characteristics.

## Discussion

These findings could be used by universities, research institutes, funding agencies, and professional organizations in Canada and elsewhere. Administrators responsible for developing research capacity and productivity will be provided with a variety of models to inform the selection, planning, and implementation of mentorship activities; guidance on the resources (human, technology, financing, tools) required for research mentorship; and knowledge about mentorship as a strategy to foster KT research and practice. Administrators responsible for planning, implementing, and evaluating mentoring programs for research, education, career development, or as a professional responsibility will be provided with information about potential barriers, strategies used elsewhere, and lessons learned that can facilitate the implementation of new mentoring programs; recommendations that could be used to enhance the outcomes or sustainability of existing mentorship activities; and tools and innovative approaches identified in the course of this research, or subsequently developed as part of planned mentorship interventions or programs. For researchers interested in evaluating how mentorship design and implementation is associated with outcomes, this study offers a unique conceptual framework by which to analyze research mentorship activities; empirical knowledge to guide the evaluation of research mentorship implementation and outcomes; and a novel perspective on mentorship form and function based on evaluation in a unique setting (health care) for an innovative purpose (share knowledge about KT research and practice).

The findings may be limited because data will have been collected from a single institution, and the views of faculty may be not transferrable to those in other settings. However, we will interview researchers reflecting a range of disciplines and other characteristics, and the findings will be validated through comparison with data from the planned scoping review, and by sharing and discussing the findings with stakeholders from a variety of institutions at a one-day workshop upon conclusion of the study. Furthermore, we will use the findings to design, implement, and evaluate KT mentoring tools and strategies in our setting and elsewhere, through a series of ongoing research studies. If successful, this program of research may increase knowledge about, confidence in, and greater utilization of KT processes, and the quality and quantity of KT research, perhaps ultimately leading to better implementation and adoption of recommended health care services.

## Competing interests

The authors declare that they have no competing interests.

## Authors' contributions

ARG conceptualized and designed this study, prepared the proposal, and obtained funding. She will lead and coordinate data collection, analysis, interpretation and report writing. She will be the primary investigator to independently review and extract data from manuscripts and interview transcripts. All investigators contributed to design of the study through several meetings, teleconferences, and email correspondence. SES and ILS will oversee conduct of the study as research mentors to ARG. LP, FW, KL, and SES will assist with the conduct and analysis of the scoping review. FW, KL, MB, and SES will assist with the conduct and analysis of interviews. All investigators will assist with interpretation, report writing, and dissemination activities, including the culminating workshop. All investigators read and approved the final version of this manuscript.
